# Single bone forearm reconstruction of proximal ulna metastatic lesion: A case report

**DOI:** 10.1016/j.ijscr.2023.108259

**Published:** 2023-04-28

**Authors:** Wazzan S. Aljuhani, Abdullah M. Alanazi, Ahmed O. Edrees

**Affiliations:** a Department of Surgery, Ministry of National Guard Health Affairs, Riyadh, Saudi Arabia; b King Abdullah International Medical Research Center, Riyadh, Saudi Arabia; c King Saud Bin Abdulaziz University for Health Sciences, Riyadh, Saudi Arabia; d Department of Orthopedic Surgery, King Abdulaziz Hospital and Oncology Center, Jeddah, Saudi Arabia

**Keywords:** Oncology, Colon, Metastasis, Orthopaedic surgery, Reconstruction, Case report

## Abstract

**Introduction and importance:**

Colorectal cancer rarely metastasizes to the bones, and if so, metastasis usually occurs in the axial skeleton. We encountered a rare case of a metastatic lesion to the right ulna arising from colonic adenocarcinoma that was treated by resection of the proximal ulna and radial neck-to-humerus trochlea transposition to salvage the limb.

**Case presentation:**

A 60-year-old man previously diagnosed with colonic adenocarcinoma presented with a single bony metastatic lesion in the right proximal ulna and was referred to our clinic for assessment. After five sessions of systemic therapy, the lesion continued to grow, causing diffuse swelling and loss of elbow range of motion. Local x-rays revealed extensive destruction of the proximal ulna and soft tissue component, with subluxation of the radial head. Magnetic resonance imaging showed an extensive lesion involving the proximal half of the ulna and a large soft tissue component. After restaging, only this metastatic lesion was found. Amputation was offered to the patient for wide margin resection, but the patient refused; therefore, we performed resection of the proximal ulna, debulking of soft tissue, and radial neck-to-humerus trochlea transposition to salvage the limb.

**Clinical discussion:**

Due to the rarity of the location, no clinical standard exists regarding the surgical treatment. Radial neck-to-humerus trochlea transposition is a valid surgical reconstruction technique to salvage the limb and preserve the hand function.

**Conclusion:**

Radial neck-to-humerus trochlea transposition is an alternative elbow reconstruction technique after proximal ulna resection in cases where other options are not ideal or contraindicated. Long-term studies are recommended to assess different surgical options for treating and reconstructing proximal ulnar tumors.

## Highlights

• A rare case of metastatis to the right ulna from colonic adenocarcinoma
• Radial neck-to-humerus trochlea transposition is effective for elbow reconstruction.
• The appropriate choice of surgical treatment depends on individual patient factors.

## 1 Introduction

Colorectal cancer (CRC) is the third most common cancer in the world [1], with a high incidence in both developed and less-developed countries [2]. In well-developed countries, there has been a decrease in the incidence and mortality rate due to improved surveillance and management protocols [3].

In Saudi Arabia, CRC is the second most common type of cancer; however, it ranks as the most common cancer in Saudi males and the third most common in Saudi females [4]. Adenocarcinoma is the most common subtype of colon cancer [5]. CRC usually metastasizes to the liver and lungs but rarely to the bones. Bone metastasis usually arises from cancers in the lungs and breasts; however, it is rare from CRC [6]. Bone metastases from CRC or any other cancer are usually detected by imaging modalities such as x-rays, computed tomography (CT), magnetic resonance imaging (MRI), or bone scans, when there is bone pain or pathological fractures. Bone metastasis arising from CRC usually occurs in the pelvis and spinal region, and the most common lesion detected on imaging is osteolytic [6]. Li et al. [7] assessed the risk factors for bone metastases from CRC and identified the patterns of bone involvement. The spine and pelvis were the most common sites for metastasis, followed by the long bones. However, no metastasis was detected in the forearm, affecting the radius or ulna. Another study by Santini et al. [8] showed that the spine is the area most commonly involved in bone metastasis, followed by the pelvis. Similarly, no metastases were detected in forearm bones. Forearm metastases from CRC are therefore extremely rare. Because this condition is so uncommon, no standard treatment methods are available. As such, treatment should be individualized according to the patient factors. In this report, we present a case of metastasis to the proximal ulna with invasion of surrounding structures originating from colorectal adenocarcinoma. This case is interesting and challenging regarding the surgical treatment. Wide debulking of the soft tissue tumor and radial neck-to-humerus trochlea transposition were performed to preserve the bony architecture and soft tissue tension. This case presentation has been reported in line with the SCARE criteria [17].

## 2 Presentation of case

**Fig. 1:**
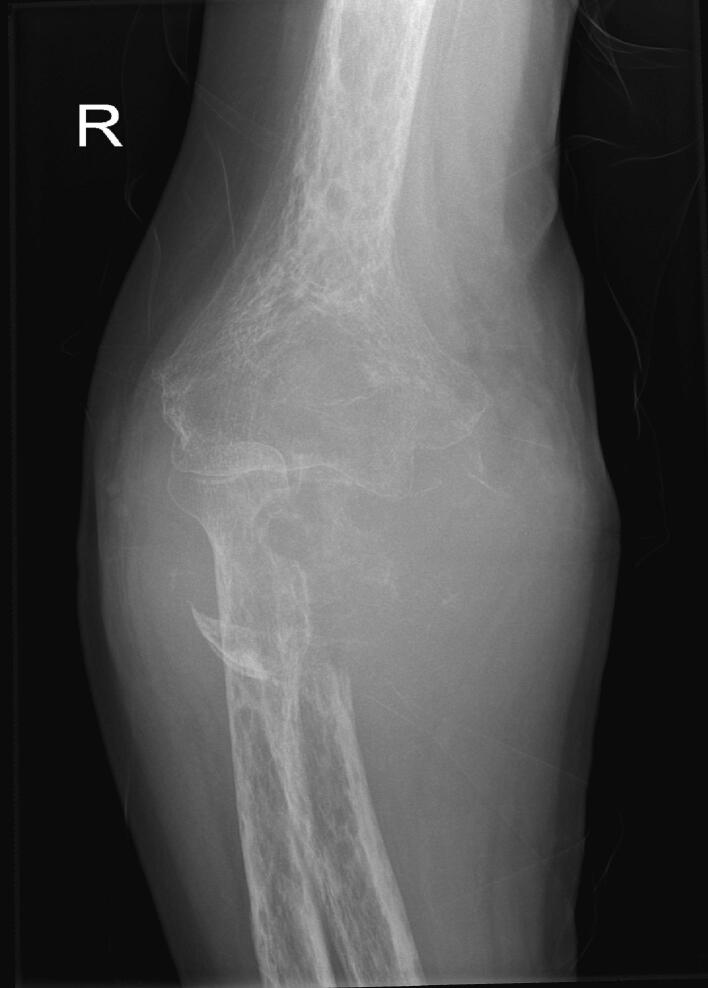
Preoperative x-ray 1.

**Fig. 2:**
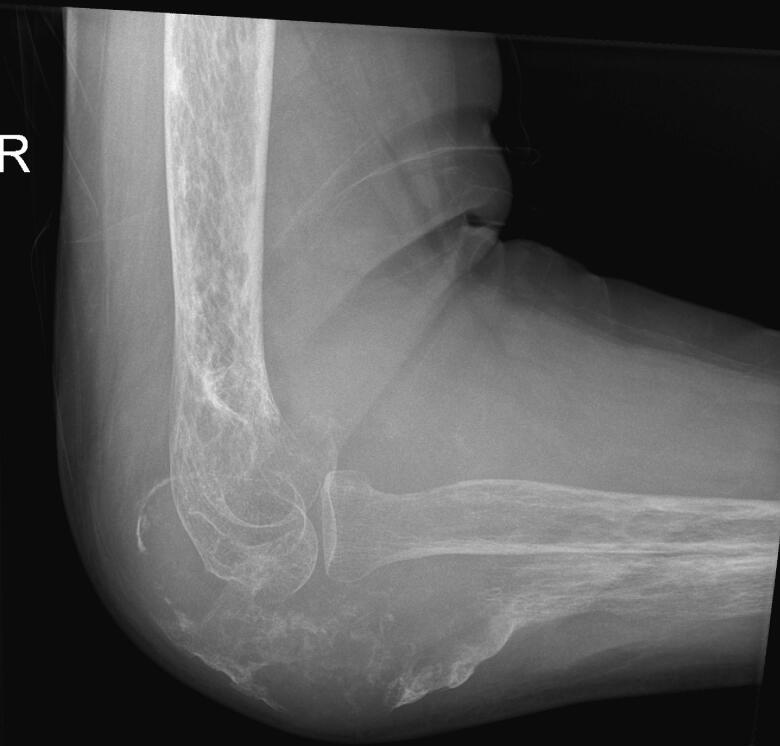
Preoperative x-ray 2.

A 60-year-old man was referred to our clinic after being diagnosed with adenocarcinoma of colonic origin with a single bony metastasis to the right ulna for which he was biopsied through core needle and diagnosed at another institution. He received systemic therapy but after 5 sessions, the metastatic lesion in the right ulna continued to progress, with growth of the soft tissue component, which caused the patient considerable pain and restriction in activity. He presented to our clinic with diffuse swelling in his proximal right forearm with almost complete loss of elbow range of motion; however, he retained hand and wrist function and range of motion. Distal pulses were intact. Local x-rays revealed extensive destruction of the proximal ulna and soft tissue component, with subluxation of the radial head (Fig. 1, Fig. 2).

**Fig. 3:**
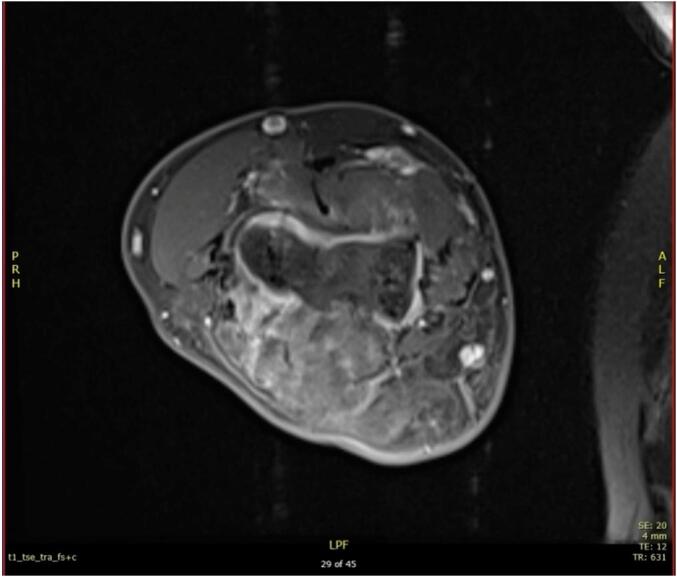
Axial section of the elbow.

**Fig. 4:**
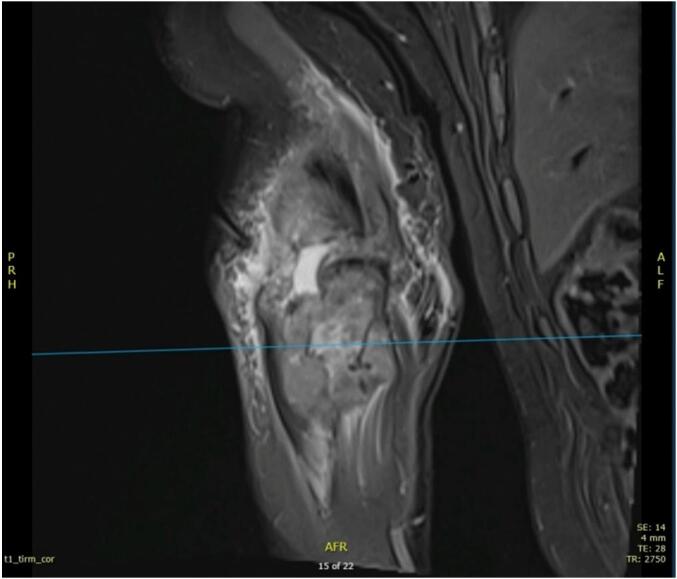
Coronal section of the elbow.

**Fig. 5:**
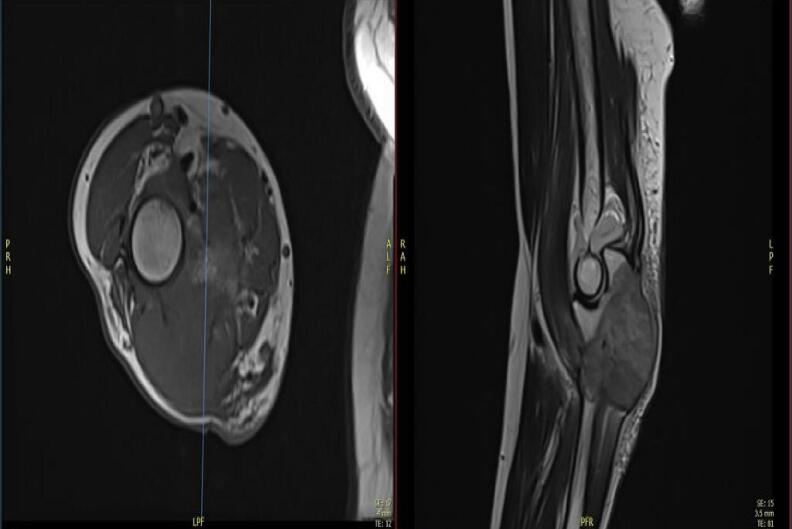
Sagittal and axial section of the elbow.

He was found to have an extensive lesion locally involving the proximal half of the ulna and a large soft-tissue component centered over the proximal half of the ulna, as shown in MRI images (Fig. 3, Fig. 4, Fig. 5).

Therefore, it was decided in conjunction with his medical oncologist to halt systemic therapy and pursue a surgical option for the metastatic lesion, after optimizing his medical condition and reviewing his laboratory results and images.

After restaging, the patient was found to have a single metastatic lesion in the proximal ulna. After discussion with his medical oncologist, it was decided that the patient has a good prognosis and to proceed with the surgery.

We waited for 2 weeks for the patient's immune system to recover. His preoperative laboratory results were within the acceptable ranges.

An above-elbow amputation was offered to the patient to obtain wide margin resection, but it was rejected in favor of a limb-sparing procedure although risks and benefits were explained thoroughly to the patient including risk of recurrence and future amputation if recurrence occurs. A single bone forearm reconstruction with proximal ulnar resection, soft tissue debulking, and radial neck-to-humerus trochlea transposition to preserve the bony architecture and soft tissue tension was offered and accepted by the patient.

**Fig. 6:**
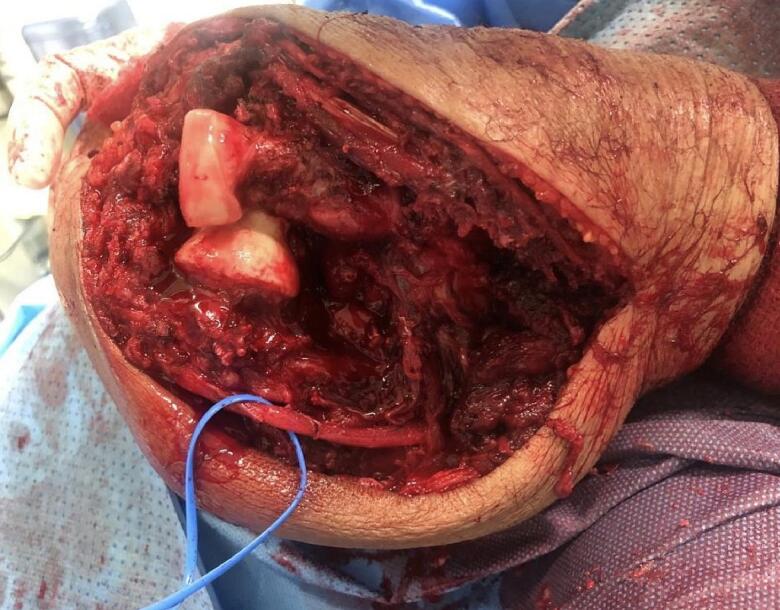
Intraoperative picture showing radial neck to trochlea transposition before reconstruction, vessel loop surrounding the ulnar nerve.

**Fig. 7:**
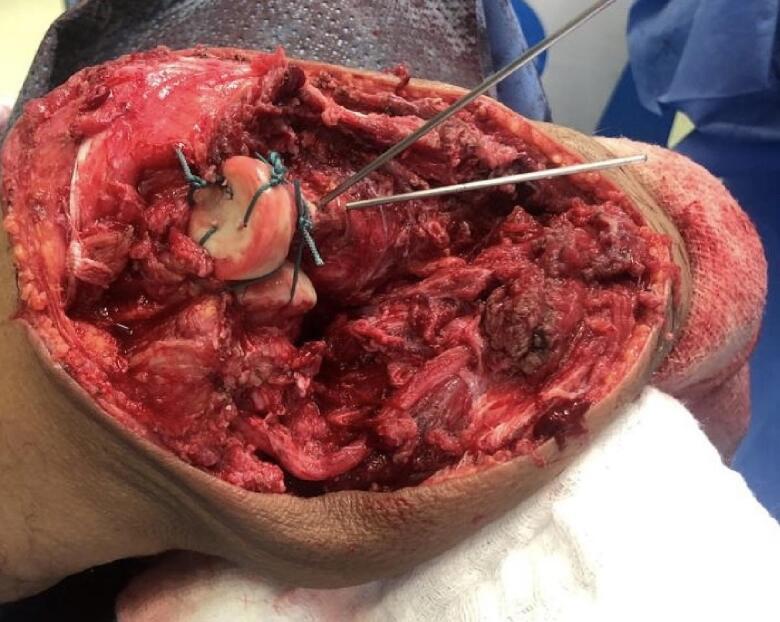
Intraoperative picture after reconstruction and application of K-wires.

**Fig. 8:**
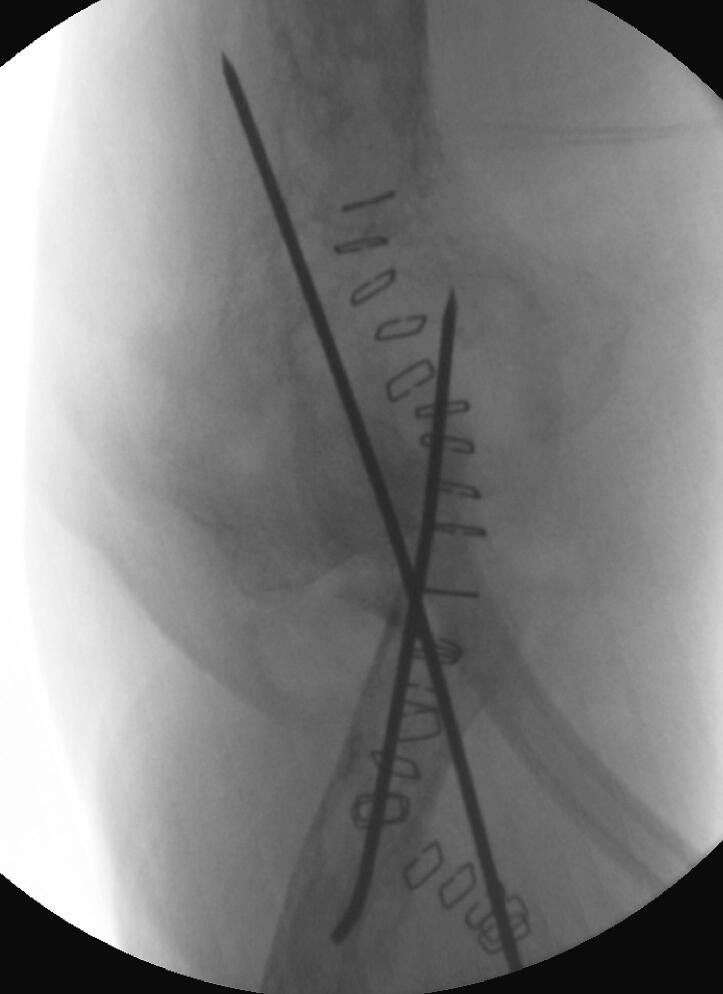
Postoperative x-ray.

The patient was positioned supine under general anesthesia, and a regional block was administered at the end of the procedure for pain control in the immediate postoperative period. A tourniquet was not used in this study. Routine prepping and draping of the affected limb, including the hand and excluding the axilla, were performed. A direct posterior ulna approach with elliptical incision around the biopsy tract was used, dissecting and protecting the ulnar nerve throughout the procedure (Fig. 6, Fig. 7). We observed that the proximal half of the ulna was involved, with an extensive soft tissue component overlapping it, thereby making it difficult to identify the plane between the flexor carpi ulnaris and extensor carpi ulnaris; this was found throughout our procedure, extending from the middle part of the ulna to the olecranon involving the distal portion of the triceps tendon. It was concluded that a negative margin would be difficult to achieve due to the invasiveness of the disease, and we proceeded with debulking the tumor in a piecemeal manner, including the most distal part of the triceps tendon. After satisfactory debulking, we moved to the ulnar osteotomy according to the preoperative templating and retrieved a frozen section from the distal resection margin, which tested negative for malignancy. At that point, we started the reconstructive part of the procedure by positioning the radial neck to the trochlear groove; due to the shortening of the triceps tendon, we elected to position the elbow at a 90° angle (functional position) and attach the remaining residual tendon to the radial head using a non-absorbable braided suture size 5 (Ethibond No. 5); to improve stability, two crossing Kirschner (K)-wires were passed through the proximal radius and the distal humerus under fluoroscopic guidance (Fig. 6, Fig. 7). The biceps tendon was involved and had to be sacrificed which was easier after resection of the proximal ulna and manipulating the proximal radius without damaging vital neurovascular structure. Closure was performed in layers and hemostasis was maintained throughout the procedure for dressing. An incisional vacuum-assisted closure (VAC) device was utilized to cover the wound, and an above-elbow splint was applied. Postoperative radiographs are shown in Fig. 8.

At 3 weeks, the wound healed; the patient had mild pain and was satisfied with the results. The patient retained hand and wrist motion and function, but no elbow range of motion was yet initiated. Subsequently, he was sent back to the medical oncologist and radiation oncology department to resume systemic therapy alongside local control in the form of radiation therapy.

At 6 weeks, the patient was seen again. The K-wires were removed at this stage of his treatment plan. The pain had improved further, and he had a stable stiff elbow and good hand and wrist function. We elected to retain the above-elbow splint for another six weeks (total 3 months), aimed at providing additional support for the elbow from radiation therapy. Patient had preserved hand function and pain with stiff elbow at 90°. Unfortunately, the patient was lost to follow-up after this.

## 3 Discussion

Bone tumors originating from primary tumors or secondary to metastasis involving the ulna are comparatively rare, although there have been cases of chondrosarcoma, osteosarcoma, primary giant cell tumors, and adamantinoma, as described previously [9]. Metastasis to the ulna is extremely rare, and if there is a secondary ulna tumor, in most cases, it originates from the kidney or lung, as it is considered an acral bone. Therefore, this poses a special challenge for surgeons in terms of surgical options. Several options have been reported, such as vascularized bone grafts, elbow arthrodesis, elbow prosthesis, radius-to-trochlea transposition, and amputation. The choice of treatment depends on patient factors, such as age, disease (primary or secondary), activities of daily living, course of treatment of the disease if the patient is on chemotherapy, radiotherapy, or both, prognosis, risk of infection, previous procedures, diabetes, increased body mass index, and patient expectations. Radius neck-to-trochlea articulation and transposition were first reported by Enneking et al. [10] Enneking credited Dr. Cable Young as the first to perform this procedure in trauma cases. Similar to our case in terms of surgical choice, Chen et al. [11] reported a case of metastasis to the proximal ulna from renal cell carcinoma treated with wide resection and reconstruction of the elbow with radius-to-trochlea articulation. The patient achieved good postoperative outcomes with a range of motion (ROM) of 10–90°; however, supination and pronation were limited. No recurrence or pathological fractures were noted at one year postoperatively. Two other cases were reported by Scott et al. [12]. The first patient achieved an acceptable outcome. The patient was able to resume most activities of daily living. His ROM was 20–140°; pronation reached 20°, and supination was restricted. During the 8-year follow-up period, no local recurrence or pathological fracture was observed. The second case was diagnosed as a recurrent soft tissue sarcoma with bony involvement of the proximal ulna. The elbow ROM was 20–130° while supination and pronation were slightly limited, in which both movements achieved 10°. There was no evidence of local recurrence or pathological fractures. Both cases were treated with wide resection and reconstruction with radius-to-trochlea transposition, and achieved acceptable outcomes. Additionally, a study applied the same procedure for treating chondrosarcoma, which resulted in 35–135° of flexion and extension; however, muscle strength was approximately half that of the normal limb [13]. Recently, two patients diagnosed with sarcoma of the proximal ulna and treated with the same procedure achieved favorable functional outcomes during two years of follow-up [14]. The aforementioned reports support the use of radius-to-trochlea transposition as a viable reconstruction option after resection of the proximal ulna tumor. However, no study has assessed the long-term outcomes of this procedure, which is critical for its adoption as a clinical standard in the treatment of proximal ulna tumors.

An alternative to radial neck-to-trochlea transposition is the use of an elbow prosthesis as an option for reconstruction after the resection of proximal ulnar tumors. Sewell et al. [15] assessed the use of endoprosthetic reconstruction of the proximal ulna following tumor resection in four young patients. One patient experienced local recurrence that required amputation, two patients exhibited flexion deformity, and one underwent revision surgery. Elbow reconstruction using a prosthesis should be individualized according to the patient's status. The patient should possess intact neuromuscular function preoperatively, because this helps to achieve good postoperative functional outcomes. In addition, the prosthesis must have sufficient soft tissue coverage. The problems associated with elbow prostheses are risk of infection, prosthesis loosening, and mechanical failure. Another factor to consider is the economic burden to the patient because in addition to cost of the procedure is the price of the prosthesis. All of the previously mentioned complications entail a risk of revision surgery, which may delay future treatment if the patient is scheduled to receive adjuvant radiotherapy, systemic therapy, or both. In our case, it was not applicable because of the risk of revision surgery in the immunocompromised state; the patient was scheduled for adjuvant radiotherapy and systemic therapy, which may delay the course of treatment to the patient, which is vital for his overall survival.

Bone grafts are considered as an option for the reconstruction of proximal ulnar defects. A case using a vascularized fibula graft to reconstruct the elbow after en bloc resection of a proximal ulnar adenoma reported that the patient achieved good functional outcomes [16]. Although the use of vascularized fibula grafts achieves favorable functional results, there are several associated problems, such as the risk of infection, non-union, donor-site morbidity, and mismatch between the ulna and fibula. In addition, the use of allografts is associated with several possible side effects, such as non-union, failure of fixation, and most importantly, infection. For each technique, to our knowledge, no study has assessed the long-term outcomes, which is the most important factor in establishing a clinical standard for reconstructing these kinds of complex cases.

In our case, the reconstructive option with the least possible side effects and probability of revision surgery, and the lowest financial impact on the patient while preserving his upper limb was considered, because the patient had CRC, and it was imperative that he return to his adjuvant treatment as soon as possible. Therefore, other surgical options were avoided because of the previously mentioned factors which would delay treatment. The most important limitation of the study is the short follow-up period, as the long-term outcomes of radius-to-trochlea transposition could not be evaluated. The aim of this surgical option was to preserve the limb, as the patient was offered amputation as a first choice, but he refused, and insisted on a procedure that could preserve his upper limb. The risk of recurrence that would entail amputation could not be eliminated because the debulking procedure is considered an intralesional resection, and the patient already has CRC.

## 4 Conclusion

Although reports of proximal ulnar tumors and their surgical options are lacking, radius-to-trochlea transposition is an alternative technique for reconstruction of the elbow after proximal ulnar resection in cases where other options are not ideal or contraindicated. The surgical options for treating proximal ulnar tumors remain controversial as they depend on multiple factors, and should be individualized to each patient's condition and requirements when possible. Further long-term studies are recommended to assess different surgical options for treating and reconstructing proximal ulnar tumors.

## Patient consent

Written informed consent was obtained from the patient for publication of this case and accompanying images. A copy of the written consent is available for review by the Editor-in chief- of this journal on request.

## Ethical approval

Ethical approval was waived by the authors institution.

## Funding

The authors received no funding for this study.

## Guarantor

Dr.Wazzan Aljuhani, Dr. Ahmed Edrees, and Dr.Abdullah Alanazi.

## CRediT authorship contribution statement

Dr. Wazzan S Aljuhani, Dr. Ahmed O Edrees, and Dr. Abdullah M Alanazi were involved in writing this case report.

## Conflict of interest

N/A.
